# The mitochondrial genomes of two *Gryllus* crickets (Grylloidea: Gryllidae) via RNA-seq

**DOI:** 10.1080/23802359.2021.2010613

**Published:** 2021-12-23

**Authors:** Alex S. Torson, Alexandra M. A. Hicks, Claire E. Baragar, David R. Smith, Brent J. Sinclair

**Affiliations:** Department of Biology, The University of Western Ontario, London, ON, Canada

**Keywords:** *Gryllus veletis*, *Gryllus lineaticeps*, RNA-seq, mitochondrial genome sequence

## Abstract

Here, we used RNA-seq reads to assemble the complete mitochondrial genomes of the spring field cricket, *Gryllus veletis*, and the variable field cricket, *Gryllus lineaticeps.* The mitochondrial genomes of *G. veletis* (15,686 bp, MW322713) and *G. lineaticeps* (15,607 bp, MW315773) each contain the expected 13 protein-coding genes, two ribosomal RNA genes, 22 transfer RNA genes, and a large control (D-loop) region. The arrangements of these features were similar for both species and consistent with other closely related Orthoptera. A phylogenetic analysis of the mitochondrial genome sequences revealed that *G. veletis* and *G. lineaticeps* cluster with the other *Gryllus* species and all reside in a clade with the Gryllidae.

Field crickets (*Gryllus* spp.; Orthoptera: Gryllidae) have a Holarctic distribution and are often subjects of evolutionary, behavioral, physiological, and acoustic studies (e.g. Adamo and Baker 2011; Blankers et al. 2017; Rodríguez-Muñoz et al. 2008). The fall field cricket, *Gryllus veletis* Alexander and Bigelow 1960, is an emerging model for insect freeze tolerance (Toxopeus et al. 2018b) that is broadly distributed in North America, whereas the variable field cricket, *Gryllus lineaticeps* Stål, 1861, featured in behavior (e.g. Wagner 1996) and physiology (e.g. Sun et al. 2020) studies, is restricted to California and Southern Oregon (Alexander and Bigelow 1960; Weissman and Gray 2019). We collected a representative *G. veletis* sample from a lab-reared colony at the University of Western Ontario, which was derived from samples originally collected from the University of Lethbridge campus in Lethbridge, Alberta, Canada (49.68° N, 112.86° W). The representative *G. lineaticeps* sample was collected from a colony maintained from field-collected individuals from Sedgwick Reserve, Santa Ynez, California, USA (34°42′N 120°1′W).

We assembled the mitochondrial genome sequences using RNA-seq reads as described by Tian and Smith (2016). Briefly, contiguous RNA sequences aligning to mitochondrial DNA sequences were mined from a *de novo* transcriptome assembly of *G. veletis* (NCBI Bioproject accession: PRJNA479659; Toxopeus et al. 2018a) using a nucleotide BLAST (blastn). The mitochondrial RNA-derived contigs were then mapped to the mitochondrial genome of *Gryllus bimaculatus* (Genbank accession: MK204367; Wang et al. 2019) using Geneious Prime v2020.2 (https://www.geneious.com). We mapped the *G. lineaticeps* reads onto the *G. bimaculatus* mitochondrial genome because a transcriptomic assembly has not yet been published. For both species, we resolved regions that did not map to the reference mitochondrial genome by remapping raw reads to the existing assembled contigs and relied on the overhang of newly mapped reads to extend the sequences until the sequence would no longer extend. We repeated this process iteratively to yield a complete mitochondrial sequence. We annotated the complete mitochondrial genomes using Geneious and tRNAscan-SE version 2.0 (Lowe and Chan 2016). The voucher specimens for *G. lineaticeps* (CNC1150967, CNC1150968, CNC1150969) and *G. veletis* (CNC1150970, CNC1150971) were deposited at the Canadian National Collection of Insects, Arachnids, and Nematodes (https://www.agr.gc.ca/eng/scientific-collaboration-and-research-in-agriculture/agriculture-and-agri-food-research-centres-and-collections/canadian-national-collection-of-insects-arachnids-and-nematodes-cnc/*;* Owen Lonsdale; owen.lonsdale@canada.ca).

The lengths of the mtDNA sequences from *G. veletis* and *G. lineaticeps* are 15,686 and 15,607 bp, respectively. These lengths are shorter than *G. bimaculatus* (16,301 bp), but comparable to related crickets in Gryllidae (e.g. *Teleogryllus emma;* 15,697 bp). Each mitochondrial genome contains the expected set of 37 genes consisting of 13 protein-coding genes (ND1-6, ND4L, COX1-3, ATP8, ATP6, and CYTB), 12S and 16S rRNAs, 22 tRNAs and a putative A + T rich (D-loop) control region. The D-loop measured 933 and 816 bp in *G. veletis* and *G. lineaticeps,* respectively. The overall base composition for *G. veletis* is A: 40.1%, C: 16.9%, G: 9.4%, and T: 33.5% and for *G. lineaticeps* is A: 39.9%, C: 17.8%, G: 9.7%, and T: 32.7%. These distributions are similar to all of the species used in our comparative analysis.

We compared the phylogenetic relationships of the two newly sequenced mitochondrial genomes with those of eight other Orthoptera presented by Wang et al. (2019) (Figure 1). We constructed the phylogenetic relationships among these species using the maximum likelihood method in Geneious. We confirmed that *G. veletis* and *G. lineaticeps* are clustered with *Gryllus bimaculatus* (Wang et al. 2019) and rooted with other Gryllidae species (*Loxoblemmus equestris, Acheta domesticus, Velarifictorus hemelytrus,* and *Teleogryllus emma*). Consistent with the nuclear DNA phylogeny published by Gray et al. (2020), *Gryllus* crickets appear to be more closely related to *Teleogryllus* than to *Acheta*.

**Figure 1.**
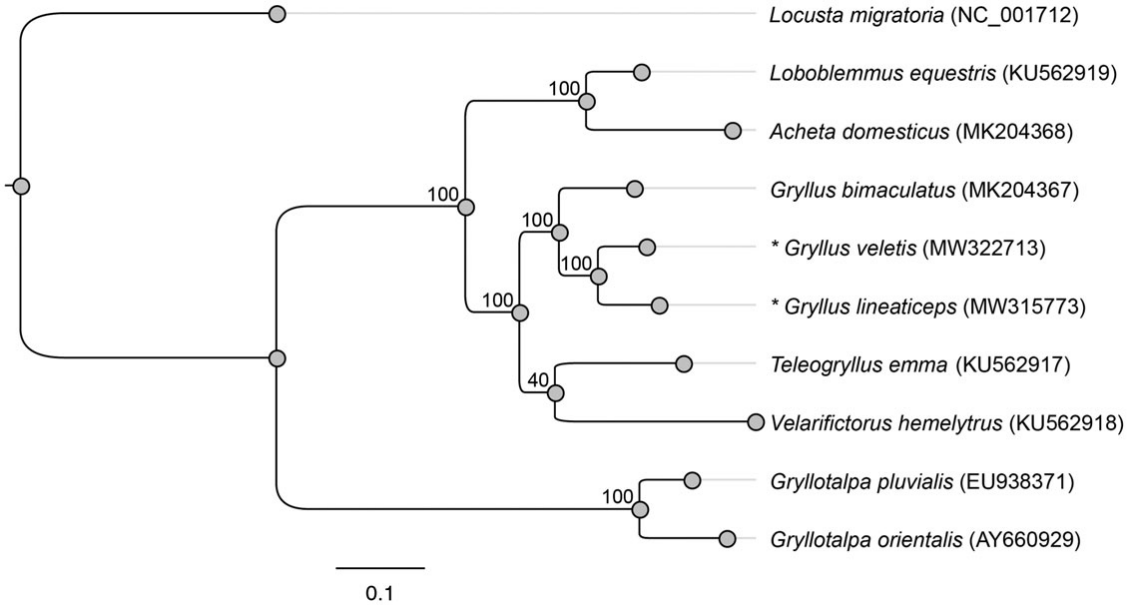
Phylogenetic positions of *Gryllus veletis* and *Gryllus lineaticeps* based on the complete mitochondrial genomes of seven other Orthoptera constructed using maximum likelihood. The numerical values indicate bootstrap support for each node (100 permutations). Each Latin name is followed by the respective mitochondrial genome GenBank accession number. The focal species of this study are denoted with an asterisk.

## Data Availability

The complete mtRNA sequences for *G. veletis* (MW322713) and *G. lineaticeps* (MW315773) are available through GenBank of NCBI at [https://www.ncbi.nlm.nih.gov]. The associated BioProject, SRA, and Bio-Sample numbers are PRJNA479659, SRP151981, and SAMN09598196-SAMN09598198, respectively.
